# miRNA therapeutics in cardiovascular diseases: promises and problems

**DOI:** 10.3389/fgene.2015.00232

**Published:** 2015-06-30

**Authors:** Nazila Nouraee, Seyed J. Mowla

**Affiliations:** Department of Molecular Genetics, Faculty of Biological Sciences, Tarbiat Modares University, TehranIran

**Keywords:** miRNA, cardiovascular disease, cardio-miR, exosome, cell communication, delivery vehicle, secretory miRNA, miRNA therapeutics

## Abstract

microRNAs (miRNAs) are a novel class of non-coding RNAs which found their way into the clinic due to their fundamental roles in cellular processes such as differentiation, proliferation, and apoptosis. Recently, miRNAs have been known as micromodulators in cellular communications being involved in cell signaling and microenvironment remodeling. In this review, we will focus on the role of miRNAs in cardiovascular diseases (CVDs) and their reliability as diagnostic and therapeutic biomarkers in these conditions. CVDs comprise a variety of blood vessels and heart disorders with a high rate of morbidity and mortality worldwide. This necessitates introduction of novel molecular biomarkers for early detection, prevention, or treatment of these diseases. miRNAs, due to their stability, tissue-specific expression pattern and secretion to the corresponding body fluids, are attractive targets for cardiovascular-associated therapeutics. Explaining the challenges ahead of miRNA-based therapies, we will discuss the exosomes as delivery packages for miRNA drugs and promising novel strategies for the future of miRNA-based therapeutics. These approaches provide insights to the future of personalized medicine for the treatment of CVDs.

## Introduction

Among multifactorial diseases, cardiovascular diseases (CVDs) are significant due to their variable symptoms and high mortality rate accounting for one third of global deaths (Santulli, 2013). They include a wide range of disorders connected to blood vessels and heart ranging from coronary artery disease (CAD), pulmonary arterial hypertension (PAH), and congenital heart disease to deep vein thrombosis and cerebrovascular disease. Major CVD risk factors include family history, obesity, hypertension, diabetes mellitus, and hypercholesterolemia (Mendis et al., 2011). Since CVDs are hard-to-cure, several investigations have focused on different mechanisms underlying CVD in order to manage the symptoms. microRNAs have emerged as one of the most favorable molecular targets in this regard. microRNAs are a class of non-coding RNAs with a short length of 18–24 nucleotides. They mainly act as post-translational repressors of gene expression. By regulating the fundamental cellular mechanisms such as cell differentiation, proliferation, growth, and apoptosis, miRNAs have received enormous attention for therapeutic applications. Huge investigations and investments have been made to bring these molecules into the clinic. In this review, we will explain the novel diagnostic, therapeutic and prognostic approaches based on miRNAs in the field of CVDs (cardio-miRs) and the problems ahead of this research area; where we are and where we expect to be in the future.

## miRNA Mechanisms of Action

Calin et al. (2002) were first to link miRNAs with cancer progression. Since then several studies have focused on the role of these small molecules in the pathogenesis of different human diseases. The primary findings emphasized on the post-transcriptional regulatory role of miRNAs through base pairing with the 3′ untranslated region (UTR) of their target mRNAs. This leads to the mRNA degradation or translation inhibition and in both cases the miRNA binding results in the suppression of their target mRNAs. This is the main mechanism reported for the miRNAs regulatory role; however, many variations have also been reported (Ha and Kim, 2014; Lin and Gregory, 2015). miRNA binding to the 5′ UTR of transcripts has also been demonstrated to be able to activate or suppress target genes (Lee et al., 2009). Some miRNAs can also bind to the open reading frame (ORF) of mRNA transcripts and repress translation. This mechanism was first reported by Tay et al. (2008). They demonstrated that miRNA binding to the coding region of pluripotency genes can regulate the embryonic stem cell differentiation. miRNA binding might also happen at the promoter of target gene which causes repression of gene translation. On the other hand, some miRNAs modulate their target expression by binding to RNA-binding proteins that regulate the expression of mRNA transcripts (Eiring et al., 2010). Salmena et al. (2011) proposed the competing endogenous RNA (ceRNA) hypothesis according to which, miRNAs, long non-coding RNAs (lncRNAs) and target mRNAs are in a finely tuned interaction through miRNA response elements (MREs) and their competition for target binding based on their total concentration in the cytoplasm defines a higher level of regulation. Recent studies have indicated another fascinating aspect of miRNA regulatory network. Fabbri et al. (2012) proved that miRNAs excreted from cancer cells, can directly bind the toll-like receptors (TLRs) at the surface of neighboring immune cells and activate the relevant signaling pathways in the recipient cells. The complexity of miRNA-mediated regulatory systems highlights the importance of these small molecules in the clinic and needs further proceeding technologies to get closer to clinical therapies.

## miRNA Diagnostics in CVD

microRNAs show tissue-specific and time-dependent expression patterns turning them into a leading fact in using these small molecules as diagnostic and therapeutic targets (Lu et al., 2005). Similar to other developmental phenomena in embryogenesis, miRNAs play critical roles in cardiovascular development and also their expression profile changes according to different pathological conditions. Cardiac resynchronization therapy (CRT) after heart failure helps improve the heart arrhythmia and it also affects the myocardial miRNA expression. In responder patients, CRT affects cardiac processes including cardiac fibrosis, apoptosis, angiogenesis, and channel alterations and consequently alters the expression levels of miR-29 (implicated in cardiac fibrosis), miR-30, miR-92, and miR-145 (involved with cardiac angiogenesis), miR-30 (modulated in cardiac apoptosis), and miR-26 (affected by modified ionic channel function; Sardu et al., 2014).

Recently circulatory miRNA expression profiling has provided strong molecular markers for detection of various diseases including CVD such as myocardial hyperthrophy, infarction, angiogenesis and fibrosis (Charan Reddy, 2014; Wronska et al., 2015). van Rooij et al. (2006) defined a miRNA signature for cardiac hypertrophy. They showed that miR-195 overexpression is sufficient to drive cardiac hypertrophy. miR-1 family, miR-133a/b and miR-208 are well-known for their implication in myogenesis both in skeletal muscles and cardiac development and their dysregulation has been detected in several CVDs including myocardial infarction, hypertrophy, and arrhythmias (Thum et al., 2008a). While miR-208 showed significant upregulation (pro-hypertrophic), miR-1 and miR-133a expression levels significantly decreased (anti-hypertrophic) in acute myocardial infarction (AMI) compared with normal individuals heart (Bostjancic et al., 2010). In CAD, miR-1 is upregulated in the left ventricular endocardium (Yang et al., 2007) and also plasma levels of miR-1, miR-133, and miR-208b have been reported to be elevated after AMI (Widera et al., 2011; Devaux et al., 2015). However, the circulatory miR-1 is not sensitive or specific enough to be known as an AMI specific biomarker since it is also affected by factors other than AMI. Moreover, by targeting Protein phosphatase 2A regulatory subunit B56 alpha (*PP2A*), miR-1 has been linked to the arrhythmia mainly through hyperphosphorylation of ryanodine receptor (RyR2; Terentyev et al., 2009). Following hypertension or cardiac infarction, cardiac hyperthrophy, and cardiac fibrosis are common diseases in which the excess amounts of extracellular matrix (ECM) proteins accumulate in cardiac tissue in order to adapt the system to the pathological conditions. Overexpression of miR-29 and miR-21 and downregulation of miR-133 and miR-30 have been linked to ECM remodeling and fibrosis by regulating several components of the ECM including collagen type I alpha 1 and 2 (*Col1A1* and *Col1A2*) as targets of miR-29 (van Rooij et al., 2008), sprouty homolog 1 (*SPRY1*) as a target of miR-21 (Thum et al., 2008b) and connective tissue growth factor (*CTGF*) as target of miR-133 and miR-30c (Duisters et al., 2009). miR-133 is specifically expressed in cardiomyocytes while miR-30 is also detectable in cardiac fibroblasts as well as cardiomyocytes. These and other miRNAs (including miR-328) have been linked to atrial fibrillation which is mainly resulted from structural remodeling and fibrosis (Santulli et al., 2014a). **Table 1** summarizes a list of miRNAs involved in different CVDs (cardio-miRs).

**Table 1:** List of cardio-miRs involved with different cardiovascular disorders.

miRNA name	Diseases name	Detection approach	Dysregulation	Reference
miR-208a/b, miR-499	AMI and myocardial injury	Microarray and real-time PCR	Upregulation in the plasma of patients	Corsten et al. (2010), Wang et al. (2010)
miR423-5p	Heart failure	Microarray and real-time PCR	Upregulation in the plasma of patients	Tijsen et al. (2010)
miR-328	Atrial fibrillation (adverse electrical remodeling)	Microarray and real-time PCR	Upregulation in left atrial samples (from dogs)	Lu et al. (2010)
miR-1	AMI	Real-time PCR	Upregulation in the patients’ serum	Cheng et al. (2010)
miR-26a	Cardiovascular repair; acute coronary syndromes	Real-time PCR	Upregulation in the Patients’ plasma	Icli et al. (2013)
miR-16, miR-27a, miR-101, and miR-150	Left ventricular contractility (LV)	Real-time PCR	Downregulation of miR-101 or miR-150 and upregulation of miR-16 or miR-27a correlate with higher risk of impaired LV contractility	Devaux et al. (2013)
miR-133a	AMI and coronary artery stenosis	Real-time PCR	Upregulation in the Patients’ plasma	Wang et al. (2013)
miR-126, miR-17/miR-92a, miR-155	Coronary artery disease	Microarray and real-time PCR	Reduction in serum and plasma of patients	Fichtlscherer et al. (2010)
miR-126	Congestive heart failure;	Real-time PCR	Negative correlation with NYHA class; downregulation in patients; positive association with incident MI	Fukushima et al. (2011)
miR-126	Myocardial infarction (MI)	Real-time PCR	Upregulation in plasma samples of MI patients; positive association with MI incidence	Zampetaki et al. (2012)
miR-1, miR-134, miR-186, miR-208, miR-223, and miR-499	AMI and angina pectoris	Deep sequencing	Upregulation in serum samples of AP patients compared to AMI	Li et al. (2013)
miR-106b/25 cluster, miR-21/590-5p family, miR-126*, miR-451	Coronary artery disease	Microarray and real-time PCR	upregulation in plasma of patients with unstable angina	Ren et al. (2013)

*AMI, acute myocardial infarction; AP, angina pectoris; NYHA, New York Heart Association.*

## miRNA-Bearing Exosomes as Communicators of CVD

Recent miRNA-based studies have focused on the role of exosomes as natural delivery vehicles for some proteins, mRNA, and miRNAs which facilitate communication between cells and their neighboring stroma. Exosomes are small vesicles (40–100 nm) originating from the plasma membrane or multivesicular bodies (MVBs) and are present in almost all biological fluids. They shuttle between neighboring cells and transfer their cargoes which can be cellular components including proteins, mRNA, and non-coding RNAs. These cargoes can perform regulatory effects and control gene expression in the recipient cells (Pegtel et al., 2010). Exosomes are famous for being the communicators of microenvironment. Exosomal membrane proteins as well as other components are usually similar to their originating cell. Their membranes usually contain higher levels of sphingomyelin, cholesterol and phosphatidylserine compared to their originating cells. Their cargo also might represent the cellular composition of RNAs and proteins or they might show a separate profile. Methodologies including immunoblotting, affinity extraction into magnetic beads and flow cytometry have been used to identify the protein components of exosomes. Profiling of RNAs – miRNAs in particular – is usually performed by means of microarrays, qPCR-based arrays and most recently by next generation sequencing techniques which analyze the transcriptome – miRnome – of each sample.

Recent studies have proved targeted packaging of miRNAs and their biogenesis components including Dicer and AGO2 in exosomes (Melo et al., 2014). Exosome shuttling is implicated in a variety of disease including cancer, CVDs or viral infections. In CVD, exosomal transfer of miRNAs is a well-known mechanism through which, cells educate their adjacent environment in order to confront the pathological condition. In heart ischemia or fibrosis, paracrine or endocrine secretion of miRNA-bearing exosomes into cardiomyocytes or active fibroblasts of the heart have been demonstrated. This leads to trans-differentiation of fibroblasts into an active state with a different rate of growth factors secretion (van Rooij and Olson, 2009; Yoo et al., 2011). Bang et al. (2014) proved that following heart infarction, exosome-secretion from cardiomyocytes with a new miRNA profile, can reprogram the cardiac fibroblasts leading to cardiac hypertrophy. These mechanisms can introduce potential novel therapeutic targets. As well, recognizing tissue-specific CAF markers, the mechanisms underlying their reactivation and the miRNA signals in these processes, will potentially provide promising targets for CVD therapies. In addition to the fibroblasts in the microenvironment, exosomal transfer of signals is also used for cardiovascular protection after a disease. For example in heart ischemia, secretory exosomes with miRNA cargos from cardiomyocytes have been shown to activate and induce the bone marrow-derived stem cells. These latter cells release the second subset of exosomes that lead to myocardial regeneration or protection (Sahoo and Losordo, 2014). Several investigations have demonstrated the selective packaging of miRNAs in exosomes and their secretion to the stroma of cells by means of signal molecules. This is beyond shedding the vesicles by plasma membrane (Yang et al., 2011; Montecalvo et al., 2012; Stoorvogel, 2012). These disease-specific expression patterns of exosomal miRNAs – which can be non-invasively detected in the body fluids – provides potential molecular markers and promising therapeutic targets for treatment of CVDs. Several circulating miRNAs have been linked to some CVDs. In patients with AMI and myocardial injury, higher levels of miR-208a have been detected in the plasma of patients and they showed higher specificity and sensitivity than conventional biomarkers such as cardiac troponin I (TnI) for diagnosis of the disease (Ji et al., 2009; Wang et al., 2010). Another potential biomarker for AMI is reported by Adachi et al. (2010) who detected higher plasma levels of miR-499 in patients with AMI compared with normal individuals. Using microarray analysis of exosomes obtained from cardiac progenitor cells, Gray et al. (2015) introduced 11 miRNAs significantly up-regulated in response to hypoxic conditions while miR-292 showed the highest variations in these exosomes. Moreover, exosome profiling from cardiac fibroblasts was shown to be enriched with miR-21-3p (miR-21*). These fibroblasts affect cardiomyocytes and secrete the mediators of cardiac hypertrophy (Bang et al., 2014). Knowing the mechanisms underlying these communications and recognizing potential strong biomarkers for diagnosis and treatment of CVDs will help us in development of future molecular therapeutics.

## miRNA Therapeutics in CVD

In the past decade, outstanding researches in the field of miRNA drugs have changed the face of molecular medicine. miRNA-based therapeutics mainly focus on reinstating the miRNA expression levels. Two main approaches include overexpression of downregulated miRNAs and suppression of overexpressed ones. These approaches have been subject to different modifications in order to improve the efficiency of delivery and less off-target effects. Promising tools in this regard are the oligonucleotides that mimic the endogenous miRNA or suppress the mature miRNA by sequence complementarity. Addition of locked nucleic acids (LNAs) or 2′-*O*-methylation of the antisense oligonucleotides increases the binding specificity while cholesterol conjugation enhances the circulation time, serum stability, and cellular uptake. Expression vectors bearing the pre-miRNA sequences or tandem miRNA target sites (sponges) sound promising for *in vitro* overexpression or suppression of miRNAs, respectively. Another issue to overcome is the efficient delivery of each of the miRNA drugs. Naked oligonucleotides are less efficient due to their instability *in vitro* or *in vivo* which subject them to different nucleases. Lipid-based vehicles, viral systems, and cationic polymers are among the main delivery tools for miRNA-based therapeutics. Each of these strategies has its own challenges and still needs improvements to address problems such as cytotoxicity, immunogenicity, and low efficiency (van Rooij and Olson, 2012). Fiedler et al. (2011) compared the effect of different doses of cholesterol-based anti-miR-24 (antagomirs) on miR-24 expression in cardiomyocytes and cardiac endothelial cells. They showed a cell-type specific tendency or mechanism for antagomir uptake by these tissues. Cholesterol-based antagomirs for silencing miR-21 were also proved to inhibit cardiac fibrosis and dysfunction (Kumarswamy et al., 2012). van Rooij et al. (2008) used anti-miR-29b oligonucleotides (cholesterol modified) after myocardial infarction and observed upregulation of ECM proteins leading to cardiac fibrosis. Also, Bonauer et al. (2009) demonstrated that intravenous injection of miR-92a antagomir, improved the function of damaged tissue in models of myocardial infarction. Inhibition of miR-92a results in neoangiogenic effects and functional recovery of ischemic tissues (Bonauer et al., 2009). Another promising miRNA in CVD therapeutics is miR-208 which is implicated in cardiac remodeling. LNA-modified anti-miR-208 oligonucleotides have successfully prevented pathologically associated cardiac remodeling during diastolic heart disease (van Rooij et al., 2007). A common issue in these approaches is *in vivo* instability and the low homing efficiency of oligonucleotides which results in modest changes in the expression levels of the target protein. Accordingly, improved stabilization or efficient delivery vehicles are required. Examples of such vehicles are adeno-associated viruses (AAVs). AAV9, a specific serotype of AAVs, has tropism for myocardiocytes and enriches in the heart (Bish et al., 2008). In another study Santulli et al. (2014b). proved that miR-126-3p down-regulation using an adeno-viral vector containing miR-126-3p target sites inhibits proliferative vascular smooth muscle cells and prevents restenosis in animal models. miR-126 is an endothelial specific miRNA that regulates vascular integrity and angiogenesis. These strategies provide an efficient tool for cardiac gene transfer and targeted delivery of miRNA-based therapeutics.

Due to their natural role in miRNA secretion and shuttling between different cells, exosomes are of great interest in miRNA therapeutics. Exosomes are flexible in size and cargo type and their non-synthetic nature potentiates them for more efficient and non-immunogenic delivery of cargo while they maintain the cargo integrity and stability. Exosomal membranes contain certain proteins which have binding affinity to specific receptors on the surface of recipient cells. So, they can selectively target cell types of interest and manipulating their miRNA components – as well as other molecular cargoes – will provide promising tools for the future of personalized medicine. In CVD, exosomes can be used as therapeutic agents, as protein delivery carriers or as gene therapy devices. Mesenchymal stem cell- derived exosomes have been investigated in the field of cardiac regenerative medicine especially in myocardial ischemia/reperfusion injuries (Lai et al., 2010). Martinez et al. (2006) have demonstrated that exosomes bearing differentiation signals can affect neovascularization and they are promising for treatment of angiogenic defects. Moreover, several exosomal miRNAs are implicated in angiogenesis and vascular repair. Zhang et al. (2010) showed that exosomes enriched with pre-miR-150 enhance endothelial cell migration. They transfected pre-miR-150/anti-miR-150 oligonucleotides to the THP-1 cell line and collected the conditioned media of these cells which was enriched with the exogenous oligonucleotides-bearing exosomes (Zhang et al., 2010). This conditioned medium affects the migration ability of endothelial cells. Exosome are novel promising elements for the future of CVD treatment due to their targeted delivery capacity and their microenvironment-dependent nature. The latter property triggers their activation in relation to the pathological microenvironment such as pH or substrate concentration. However, further studies would be necessary to overcome obstacles such as engineering and purifying exosomes, cargo loading into them and optimizing their quality and characterization for targeted delivery.

## Conclusion

Cardiovascular diseases are the one of the leading causes of death worldwide. Most of CVDs can be prevented by controlling behavioral and environmental risk factors and early diagnosis and management play an important role in this regard. In this review, we focused on miRNAs as small non-coding RNAs involved in a variety of key cellular processes. Several miRNA biomarkers have been introduced for different CV situations. miRNAs have emerged as attractive novel therapeutics and they have several advantages over other molecular therapeutics due to their small size, conserved sequences and their stability in the body fluids. First anti-cancer miRNA-based drug, MRX-34 (a liposome-based miR-34 mimic) developed by Mirna Therapeutics came to the clinic in 2013 for the treatment of hepatocellular carcinoma (mirnarx.com; NCT01829971). Current achievements have portrayed a promising future for miRNA-based therapeutics although still several obstacles including their stability, renal clearance, off-target effects, inefficient endocytosis by target cells or the immunogenicity of delivery vehicles, need to be overcome. In CVD, some miRNA-based strategies have resulted in promising findings, including anti-miR-126 approaches that resulted in vascular smooth muscle cells and restenosis inhibition (Santulli et al., 2014b). Also, novel methodologies such as exosome-based delivery of miRNA drugs provide reliable evidence to overcome impediments such as inefficient, unspecific delivery, and immunogenic reactions. But these drugs still need investigations on different aspects of miRNA biology, their long term effects, subsequent biochemical and off-target effects and miRNA pathway analysis.

## Conflict of Interest Statement

The authors declare that the research was conducted in the absence of any commercial or financial relationships that could be construed as a potential conflict of interest.
